# Point-of-Care Ultrasound (POCUS) in Shock Diagnosis: Perceptions, Usage, and Barriers Among Emergency Physicians in Riyadh, Saudi Arabia

**DOI:** 10.7759/cureus.112614

**Published:** 2026-07-13

**Authors:** Manal A Abudeyah, Nouf A Alsadoun, Hadeel M Alomrani

**Affiliations:** 1 Emergency Department, Prince Sultan Military Medical City, Riyadh, SAU

**Keywords:** emergency medicine, emergency physicians, point-of-care ultrasound, saudi arabia, shock, ultrasonography

## Abstract

Background

Point-of-care ultrasound (POCUS) has become an important bedside tool for the rapid assessment of patients presenting with shock. However, data on its utilization and barriers among emergency physicians in Saudi Arabia remain limited.

This study aimed to evaluate the frequency of POCUS use, physicians’ perceptions, and barriers to its implementation in shock diagnosis among emergency physicians in Riyadh.

Methods

A cross-sectional survey was conducted among emergency medicine consultants, specialists, and senior residents practicing in Riyadh, Saudi Arabia. Participants were recruited between October 2025 and March 2026 using convenience and snowball sampling. The questionnaire assessed demographics, POCUS utilization, perceptions, benefits, barriers, and associated factors. Descriptive and inferential statistical analyses were performed using Stata version 18 (StataCorp, College Station, TX).

Results

A total of 152 emergency physicians were included in the analysis. Frequent POCUS use (daily or 3-5 times/week) was reported by 96 (63.2%) participants. The Rapid Ultrasound in SHock (RUSH) protocol was the most commonly used shock assessment protocol, reported by 137 (90.1%) participants. Most participants agreed that POCUS improves diagnostic accuracy (138 (90.8%)), reduces clinical decision-making time (132 (86.8%)), and should be a mandatory skill in emergency medicine training (142 (93.4%)). Faster diagnosis was identified as the most important benefit by 141 (92.8%) participants. The most commonly reported barriers were time constraints (93 (61.2%)), limited access to ultrasound machines (82 (54.0%)), and lack of formal training (78 (51.3%)). Frequent POCUS use was significantly associated with hospital type (χ² = 22.279, p < 0.001) and employment in a tertiary care center (χ² = 4.701, p = 0.030). Higher perception scores were associated with frequent POCUS use (z = 4.460, p < 0.001) and years of emergency medicine experience (χ² = 6.948, p = 0.031).

Conclusion

POCUS is widely utilized and highly valued by emergency physicians in Riyadh for the assessment of shock. Despite favorable perceptions, barriers related to training, equipment availability, and time constraints remain common. Structured training and institutional support may facilitate broader POCUS integration into emergency practice.

## Introduction

Shock is a time-sensitive medical emergency characterized by inadequate tissue perfusion and impaired oxygen delivery, leading to progressive organ dysfunction if not recognized and treated promptly. It represents one of the most challenging conditions encountered in emergency departments (EDs), where rapid identification of the underlying cause directly influences treatment decisions and patient outcomes [1,2]. Because different types of shock, including hypovolemic, distributive, cardiogenic, and obstructive shock, require different management strategies, delays in establishing the correct diagnosis may increase morbidity and mortality [3].

Traditionally, the initial evaluation of patients with shock has relied on clinical examination, laboratory investigations, and advanced imaging modalities. While these approaches remain important, they often require time and may not provide immediate answers during the critical early stages of resuscitation. In contrast, point-of-care ultrasound (POCUS) enables emergency physicians to obtain real-time bedside information regarding cardiac function, intravascular volume status, pulmonary pathology, and major vascular abnormalities without interrupting patient care [4,5]. This immediate bedside information may help clinicians narrow the differential diagnosis and guide early management decisions.

Structured point-of-care ultrasound protocols, such as the Sonography in Hypotension and Cardiac Arrest (SHoC) protocol, provide a systematic framework for the bedside evaluation of patients with undifferentiated hypotension and shock [6]. Previous studies have shown that POCUS is associated with improved diagnostic confidence and more timely clinical decision-making in critically ill patients and during shock assessment [7,8]. However, POCUS is intended to complement, rather than replace, comprehensive clinical assessment and advanced imaging when clinically indicated.

Despite these advantages, routine implementation of POCUS remains inconsistent across healthcare systems. Reported barriers include limited formal training, inadequate access to ultrasound equipment, lack of standardized institutional protocols, and variations in physician confidence and competency [9,10]. These factors may reduce the frequency with which POCUS is incorporated into the evaluation of patients presenting with shock, even in institutions where ultrasound devices are available.

In Saudi Arabia, emergency care is delivered across the Ministry of Health, military, university-affiliated, and private hospitals, where the availability of ultrasound equipment, institutional support, and physician training may vary. Previous Saudi studies have primarily focused on POCUS education, residency training, and physician competency. However, limited evidence is available regarding the routine clinical use of POCUS for shock assessment, emergency physicians’ perceptions, and perceived barriers to its implementation in daily emergency practice, particularly in Riyadh. Addressing this gap may provide valuable insight into current practice patterns and help inform future educational initiatives, resource planning, and research related to POCUS implementation in emergency departments.

The primary objective of this study was to evaluate the frequency of POCUS use during shock assessment among emergency physicians working in Riyadh, Saudi Arabia. The secondary objectives were to assess physicians’ perceptions of the clinical value of POCUS and identify perceived barriers associated with its implementation in routine emergency practice.

## Materials and methods

Study design and participants

A cross-sectional survey was conducted among emergency medicine physicians practicing in Riyadh, Saudi Arabia. The study included emergency medicine consultants, specialists, and senior residents (postgraduate year 3 (PGY-3) and postgraduate year 4 (PGY-4)) working in the Ministry of Health, military, university-affiliated, and private hospitals. Demographic and professional information was collected, including age, gender, years of experience in emergency medicine, current position, type of hospital, and whether participants were practicing in a tertiary care center.

Sampling and data collection

Consistent with previous survey-based studies conducted among emergency physicians in Riyadh, this study used a non-probability convenience sampling approach to recruit all accessible and eligible emergency medicine consultants, specialists, and senior residents during the study period. Because no comprehensive registry of emergency physicians practicing in Riyadh was available, the total number of eligible physicians could not be determined; therefore, a response rate could not be calculated. Similar recruitment strategies have been adopted in previous local studies [11,12]. No formal sample size calculation was performed because the study aimed to recruit all accessible eligible emergency physicians during the study period using convenience and snowball sampling.

Participants were recruited through hospital department contacts, professional emergency medicine networks, and WhatsApp groups. Snowball sampling was also employed by encouraging participants to distribute the survey link to eligible colleagues practicing in different hospitals across Riyadh. This recruitment strategy was intended to maximize participation across multiple healthcare sectors; however, it may have introduced selection bias by preferentially reaching physicians with greater interest in POCUS. Participant recruitment and data collection were conducted between October 2025 and March 2026.

Data were collected using a self-administered electronic questionnaire developed using Google Forms (Google, Inc., Mountain View, CA). Participation was voluntary, and electronic informed consent was obtained before participants accessed the survey.

Prior to full-scale data collection, the questionnaire was pilot tested among 10 emergency physicians with demographic and professional characteristics similar to the target study population to assess its clarity, comprehensibility, and face validity. Based on participants’ feedback, minor wording modifications were made to improve clarity, and no substantial changes to the questionnaire content were required. The pilot participants were excluded from the final study sample. No additional reliability or validity testing beyond assessment of internal consistency using Cronbach’s alpha was performed.

A total of 154 emergency physicians completed the survey. Two respondents who did not provide informed consent were excluded, resulting in a final analytical sample of 152 participants.

Survey instrument

The questionnaire was adapted from previously published surveys evaluating point-of-care ultrasound (POCUS) utilization among emergency physicians [9]. Minor modifications were made to tailor the questionnaire to the objectives of the current study, including items assessing POCUS use specifically for shock diagnosis, perceived barriers, perceived benefits, and strategies to improve POCUS implementation in emergency medicine practice. The questionnaire comprised four domains.

The first domain collected participants’ demographic and professional characteristics, including age group, gender, years of experience in emergency medicine, current position, hospital type, and employment in a tertiary care center.

The second domain assessed POCUS utilization and practice patterns, including the frequency of POCUS use in diagnosing shock, ultrasound protocols used for shock assessment (e.g., Rapid Ultrasound in SHock (RUSH), Focused Assessment with Sonography in Trauma (FAST), and SHoC), and the clinical scenarios in which POCUS was most frequently utilized. Multiple responses were permitted where applicable.

The third domain evaluated participants’ perceptions toward POCUS using five statements rated on a 5-point Likert scale ranging from 1 (strongly disagree) to 5 (strongly agree). The statements assessed perceptions regarding diagnostic accuracy, reduction in clinical decision-making time, the need for formal POCUS training, reduced reliance on advanced imaging, and confidence in using POCUS. A composite perception score ranging from 5 to 25 was calculated by summing the responses to the five items, with higher scores indicating more positive perceptions toward POCUS. The internal consistency of the perception scale was assessed using Cronbach’s alpha coefficient, indicating excellent internal consistency (Cronbach’s α = 0.861).

The fourth domain explored participants’ perceived benefits of POCUS, barriers limiting its routine use, and potential strategies to improve its implementation in emergency medicine practice. Participants were asked to identify the perceived advantages of POCUS for diagnosing shock, barriers to its routine implementation, and recommendations for improving its adoption in emergency departments. Multiple responses were allowed for these questions.

Statistical analysis

Data were analyzed using Stata version 18 (StataCorp, College Station, TX). Descriptive statistics were used to summarize the study variables. Categorical variables were presented as frequencies and percentages, whereas continuous or ordinal variables that were not normally distributed were summarized using the median and interquartile range (IQR).

Before analysis, selected variables, including age category, hospital type, and frequency of POCUS use, were reclassified to facilitate statistical comparisons. Before inferential analyses, hospital categories with very small numbers of participants (e.g., Ministry of Interior and Security Forces hospitals) were combined with the military hospital category to ensure adequate cell counts for statistical comparisons. Participants were categorized into frequent users (daily or regularly (3-5 times/week)) and less frequent users (occasionally (1-2 times/week) or rarely (1-2 times/month)). This dichotomization was performed to facilitate statistical comparisons and ensure adequate sample sizes within comparison groups.

The internal consistency of the five-item perception scale was assessed using Cronbach’s alpha coefficient. The normality of the overall POCUS perception score was assessed using the Shapiro-Wilk test. Because the perception score was not normally distributed (Shapiro-Wilk p < 0.001), non-parametric statistical tests were used for group comparisons.

Associations between categorical variables were evaluated using the Chi-square test. The association between participant characteristics and overall POCUS perception scores was assessed using non-parametric methods. The Mann-Whitney U test was used for variables with two categories, whereas the Kruskal-Wallis test was used for variables with more than two categories. A two-sided p-value < 0.05 was considered statistically significant.

Ethical considerations

Ethical approval for this study was obtained from the Institutional Review Board (IRB) of Prince Sultan Military Medical City (approval number: E-2680) before the commencement of the study. Participation was voluntary, and electronic informed consent was obtained from all participants before completing the questionnaire. No personally identifiable information was collected, and all responses were kept confidential and stored securely. Access to the study data was restricted to the research team.

## Results

Participant characteristics

A total of 154 emergency physicians completed the survey. Two respondents who did not provide informed consent were excluded, resulting in a final analytical sample of 152 participants.

The demographic and professional characteristics of the participants are presented in Table 1. More than half of the participants were younger than 30 years (85, 55.9%), while 49 (32.2%) were aged 31-39 years and 18 (11.8%) were aged 40 years or older. Male participants accounted for 87 (57.2%) of the study population.

**Table 1:** Demographic Characteristics of the Participants (N = 152) MOH: Ministry of Health, MOI: Ministry of Interior

Characteristics	Responses	
Age group	Number	%
<30 years	85	55.92
31-39 years	49	32.24
40-49 years	16	10.53
>50 years	2	1.32
Gender		
Male	87	57.24
Female	65	42.76
Years of experience in emergency medicine		
0-5 years	94	61.84
6-10 years	35	23.03
>10 years	23	15.13
Current position		
Senior emergency medicine resident	95	62.50
Emergency medicine specialist	31	20.39
Emergency medicine consultant	26	17.11
Type of hospital		
MOH	69	45.39
Military	40	26.32
University-affiliated training center	23	15.13
Private	18	11.84
MOI	1	0.66
Security forces hospital	1	0.66
Work in a tertiary care center		
Yes	113	74.34
No	39	25.66

Most participants had 0-5 years of experience in emergency medicine (94, 61.8%), followed by 35 (23.0%) with 6-10 years of experience and 23 (15.1%) with more than 10 years of experience. Senior emergency medicine residents represented the largest professional group (95, 62.5%), followed by emergency medicine specialists (31, 20.4%) and emergency medicine consultants (26, 17.1%).

Regarding workplace characteristics, 69 (45.4%) participants worked in Ministry of Health hospitals, 40 (26.3%) in military hospitals, 23 (15.1%) in university-affiliated training centers, and 18 (11.8%) in private hospitals. Overall, 113 (74.3%) participants reported working in a tertiary care center (Table 1).

POCUS utilization and practice patterns

The frequency and patterns of POCUS utilization for shock diagnosis are presented in Table 2. The most commonly reported frequency of POCUS use was 3-5 times per week (56, 36.8%), followed by 1-2 times per week (42, 27.6%), daily use (40, 26.3%), and 1-2 times per month (14, 9.2%). Frequency refers to participants’ self-reported use of POCUS during a typical working week rather than the number of patients assessed. No participants selected the “Never” response category (n = 0); therefore, this category was not presented in Table 2.

**Table 2:** Frequency and Patterns of POCUS Use in Shock Diagnosis (N = 152) Multiple responses were allowed for the POCUS protocols and clinical conditions questions; therefore, percentages may exceed 100%. Other protocols were derived from free-text responses and included IVC only and DVT/pregnancy-related protocols. Clinical conditions classified under “Other” were derived from free-text responses and included DVT, pregnancy-related conditions, and OBGYN conditions. POCUS: point-of-care ultrasound

Variables	Responses	
Frequency of POCUS use in shock diagnosis	Number	%
Daily	40	26.32
Frequently (3-5 times/week)	56	36.84
Occasionally (1-2 times/week)	42	27.63
Rarely (1-2 times/month)	14	9.21
POCUS protocols used for shock evaluation*		
RUSH	137	90.13
FAST	82	53.95
SHoC	14	9.21
Other (IVC only, DVT/pregnancy-related protocols)	2	1.32
Clinical conditions for which POCUS is most commonly used*		
Undifferentiated shock	134	88.16
Trauma	132	86.84
Cardiac arrest	96	63.16
Pulmonary embolism	90	59.21
Hypovolemic shock	90	59.21
Cardiogenic shock	89	58.55
Sepsis	69	45.39
Other (DVT, pregnancy-related conditions, OBGYN conditions)	2	1.32

The RUSH protocol was the most commonly used protocol for shock assessment (137, 90.1%), followed by FAST (82, 54.0%) and SHoC (14, 9.2%). A small proportion of participants reported using other protocols, including IVC-only and DVT/pregnancy-related protocols (2, 1.3%).

Undifferentiated shock was the most frequently reported clinical indication for POCUS use (134, 88.2%), followed closely by trauma (132, 86.8%). Other frequently reported indications included cardiac arrest (96, 63.2%), pulmonary embolism (90, 59.2%), hypovolemic shock (90, 59.2%), cardiogenic shock (89, 58.6%), and sepsis (69, 45.4%) (Table 2).

Perceptions toward POCUS use

Participants generally reported positive perceptions regarding the use of POCUS in the management of shock (Table 3). The majority agreed or strongly agreed that POCUS improves diagnostic accuracy (138, 90.8%), reduces clinical decision-making time (132, 86.8%), and should be a mandatory skill in emergency medicine training (142, 93.4%). Most participants also reported confidence in their ability to use POCUS successfully (123, 80.9%). Perceptions regarding the ability of POCUS to reduce reliance on advanced imaging were comparatively less favorable, with 86 (56.6%) agreeing or strongly agreeing with this statement.

**Table 3:** Attitudes and Perceptions Toward POCUS Use in Shock Management (N = 152) Note: Percentages are based on the total number of respondents (N = 152) and may not sum exactly to 100% because of rounding. POCUS: point-of-care ultrasound

Statements	Responses (number (%))				
	Strongly disagree	Disagree	Neutral	Agree	Strongly agree
POCUS improves diagnostic accuracy in shock management	3 (1.97)		11 (7.24)	51 (33.55)	87 (57.24)
POCUS reduces time to clinical decision-making	3 (1.97)	3 (1.97)	14 (9.21)	55 (36.18)	77 (50.66)
POCUS should be a mandatory skill in emergency medicine training	3 (1.97)	1 (0.66)	6 (3.95)	49 (32.24)	93 (61.18)
POCUS use decreases reliance on advanced imaging (CT/MRI)	7 (4.61)	19 (12.50)	40 (26.32)	35 (23.03)	51 (33.55)
I feel confident in my ability to use POCUS effectively	5 (3.29)	3 (1.97)	21 (13.82)	64 (42.11)	59 (38.82)

Perceived benefits of POCUS

The perceived benefits of POCUS in the diagnosis of shock reported by participants are presented in Table 4. Faster diagnosis was the most frequently selected perceived benefit (141, 92.8%), followed by increased physician confidence (88, 57.9%). Participants also perceived improved patient outcomes (62, 40.8%) and a reduced need for invasive procedures (58, 38.2%) as benefits of POCUS. These findings reflect physicians’ perceptions and were not based on measured clinical or patient outcomes. Additional free-text responses highlighted improved diagnostic accuracy, facilitation of invasive procedures, and narrowing of the differential diagnosis as additional perceived advantages of POCUS.

**Table 4:** Perceived Benefits of Using POCUS in Shock Diagnosis (N = 152) Note: Participants were allowed to select more than one response; therefore, percentages do not sum to 100%. POCUS: point-of-care ultrasound, ED: emergency department

Variables	Responses	
Perceived benefits of POCUS in shock diagnosis	Number	%
Faster diagnosis	141	92.76
Increased physician confidence	88	57.89
Perceived improvement in patient outcomes (e.g., reduced mortality, shorter ED stays)	62	40.79
Reduced need for invasive procedures	58	38.16

Barriers to POCUS implementation

The perceived barriers to routine POCUS implementation are summarized in Table 5. Time constraints in the emergency department were the most frequently reported barrier (93, 61.2%), which may reflect heavy clinical workload and limited time available to perform ultrasound examinations during patient care. Limited access to ultrasound machines (82, 54.0%) may indicate that ultrasound equipment was not consistently available when needed. Lack of formal POCUS training (78, 51.3%) and lack of confidence in image interpretation (61, 40.1%) were also commonly reported barriers, whereas lack of institutional support was the least frequently reported barrier (36, 23.7%). One participant additionally reported that the limited availability of functional ultrasound machines and increased patient workload further restricted routine POCUS use.

**Table 5:** Main Barriers Preventing Routine POCUS Use in Clinical Practice (N = 152) Note: Participants were allowed to select more than one response; therefore, percentages do not sum to 100%. POCUS: point-of-care ultrasound, ED: emergency department

Variables	Responses	
Barriers	Number	%
Lack of formal training	78	51.32
Lack of confidence in interpretation	61	40.13
Limited access to ultrasound machines	82	53.95
Time constraints in the ED	93	61.18
Lack of institutional support (e.g., funding, administrative policies, mentorship)	36	23.68
Other (lack of functional, easy-to-use ultrasound machines, increased patient load)	1	0.66

Factors perceived to improve POCUS adoption

Participants identified several strategies that could improve the adoption of POCUS in emergency medicine practice (Table 6). Mandatory residency training was the most frequently selected strategy (113, 74.3%), followed by hands-on workshops (107, 70.4%) and increased availability of ultrasound machines (104, 68.4%). Institutional policies supporting POCUS use were selected by 61 (40.1%) participants.

**Table 6:** Perceived Factors for Improving POCUS Adoption in Emergency Medicine (N = 152) POCUS: point-of-care ultrasound

Variables	Responses
Factors improving POCUS adoption	Selected (number (%))
Mandatory residency training	113 (74.34%)
Hands-on workshops	107 (70.39%)
Increased availability of ultrasound machines	104 (68.42%)
Institutional policies supporting POCUS use	61 (40.13%)

Factors associated with frequent POCUS use

Table 7 summarizes the associations between participants’ demographic and professional characteristics and the frequency of point-of-care ultrasound (POCUS) use in shock diagnosis. Frequent POCUS use was defined as daily use or regular use (3-5 times per week), whereas less frequent use included occasional (1-2 times per week) or rare (1-2 times per month) use.

**Table 7:** Factors Associated With Frequent POCUS Use in Shock Diagnosis (N = 152) Note: Data are presented as number (%) for categorical variables. Chi-square test was used to examine associations between categorical variables. *A p-value of less than 0.05 was considered statistically significant. POCUS: point-of-care ultrasound, MOH: Ministry of Health

Variables	Frequent (number (%))	Less frequent (number (%))	Total	Test	Test statistic	P-value
Gender				Chi-square	χ² = 0.438	0.508
Female	43 (66.15%)	22 (33.85%)	65			
Male	53 (60.92%)	34 (39.08%)	87			
Age group				Chi-square	χ² = 0.636	0.728
<30 years	56 (65.88%)	29 (34.12%)	85			
31-39 years	29 (59.18%)	20 (40.82%)	49			
≥40 years	11 (61.11%)	7 (38.89%)	18			
Years of experience in emergency medicine				Chi-square	χ² = 0.558	0.756
0-5 years	61 (64.89%)	33 (35.11%)	94			
6-10 years	22 (62.86%)	13 (37.14%)	35			
>10 years	13 (56.52%)	10 (43.48%)	23			
Current position				Chi-square	χ² = 0.446	0.8
Emergency medicine consultant	17 (65.38%)	9 (34.62%)	26			
Emergency medicine specialist	18 (58.06%)	13 (41.94%)	31			
Senior emergency medicine resident	61 (64.21%)	34 (35.79%)	95			
Hospital type				Chi-square	χ² = 22.279	<0.001*
MOH	48 (69.57%)	21 (30.43%)	69			
Private	9 (50.00%)	9 (50.00%)	18			
University-affiliated training center	22 (95.65%)	1 (4.35%)	23			
Military/other governmental	17 (40.48%)	25 (59.52%)	42			
Work in a tertiary care center				Chi-square	χ² = 4.701	0.030*
No	19 (48.72%)	20 (51.28%)	39			
Yes	77 (68.14%)	36 (31.86%)	113			

A statistically significant association was observed between hospital type and frequent POCUS use (χ² = 22.279, p < 0.001). Physicians working in university-affiliated training centers demonstrated the highest proportion of frequent POCUS users (22, 95.65%), followed by those working in Ministry of Health hospitals (48, 69.57%). In contrast, physicians working in military and other governmental hospitals reported the lowest proportion of frequent POCUS use (17, 40.48%).

Working in a tertiary care center was also significantly associated with more frequent POCUS use (χ² = 4.701, p = 0.030). Among physicians working in tertiary care centers, 77 (68.14%) reported frequent POCUS use compared with 19 (48.72%) of those working in non-tertiary care centers.

No statistically significant associations were identified between POCUS use frequency and gender (χ² = 0.438, p = 0.508), age group (χ² = 0.636, p = 0.728), years of emergency medicine experience (χ² = 0.558, p = 0.756), or current professional position (χ² = 0.446, p = 0.800). These findings suggest that hospital type and employment in a tertiary care center were associated with more frequent POCUS use in this study. However, because of the cross-sectional study design, these findings should not be interpreted as evidence of a causal relationship.

Factors associated with POCUS perception scores

Table 8 summarizes the associations between participants’ characteristics and overall POCUS perception scores. Significant differences in perception scores were observed according to years of emergency medicine experience (Kruskal-Wallis χ² = 6.948, p = 0.031) and frequency of POCUS use (Mann-Whitney U, z = 4.460, p < 0.001).

**Table 8:** Association Between POCUS Perception Total Score and Participants’ Characteristics (N = 152) Note: Data are presented as median (IQR). The Mann-Whitney U test was used for comparisons between two groups, and the Kruskal-Wallis test was used for comparisons involving three or more groups. *A two-sided p-value < 0.05 was considered statistically significant. POCUS: point-of-care ultrasound, IQR: interquartile range, MOH: Ministry of Health

Variables	Category	Number	POCUS perception total score (median (IQR))	Test	Test statistic	P-value
Gender	Female	65	21 (19-25)	Mann-Whitney U	z = 0.115	0.909
	Male	87	21 (19-24)			
Age group (years)	<30	85	21 (19-24)	Kruskal-Wallis	χ² = 0.099	0.951
	31-39	49	21 (19-24)			
	≥40	18	21 (19-25)			
Years of experience in emergency medicine	0-5 years	94	21 (19-24)	Kruskal-Wallis	χ² = 6.948	0.0310*
	6-10 years	35	24 (20-25)			
	>10 years	23	20 (18-22)			
Current position	Emergency medicine consultant	26	21 (19-24)	Kruskal-Wallis	χ² = 0.871	0.646
	Emergency medicine specialist	31	21 (20-25)			
	Senior emergency medicine resident	95	21 (19-24)			
Hospital type	MOH	69	21 (19-25)	Kruskal-Wallis	χ² = 3.913	0.271
	Private	18	21.5 (20-25)			
	University-affiliated training center	23	23 (19-25)			
	Military/other governmental	42	20 (19-23)			
Working in a tertiary care center	No	39	21 (18-24)	Mann-Whitney U	z = 0.079	0.938
	Yes	113	21 (19-24)			
POCUS use frequency	Frequent use	96	23 (20-25)	Mann-Whitney U	z = 4.460	<0.001*
	Less frequent use	56	20 (18-22)			

Participants who reported frequent POCUS use had higher perception scores than less frequent users, with a median score of 23 (IQR: 20-25) compared with 20 (IQR: 18-22). Participants with six to ten years of emergency medicine experience had the highest perception scores (median 24, IQR: 20-25), compared with participants with zero to five years of experience (median 21, IQR: 19-24) and those with more than ten years of experience (median 20, IQR: 18-22).

No significant differences in overall POCUS perception scores were observed according to gender, age group, current professional position, hospital type, or employment in a tertiary care center (Table 8).

## Discussion

The present study provides important insights into the utilization of point-of-care ultrasound (POCUS) for shock diagnosis among emergency physicians in Riyadh, Saudi Arabia. Overall, POCUS was widely incorporated into routine clinical practice, with 96 (63.2%) participants reporting frequent use in the assessment of shock.

The RUSH protocol was the predominant protocol for shock assessment, and participants demonstrated overwhelmingly positive perceptions regarding the role of POCUS in improving diagnostic accuracy and facilitating timely clinical decision-making. Nevertheless, several barriers to routine implementation were identified, particularly time constraints, limited access to ultrasound machines, and inadequate formal training. In addition, physicians who reported frequent POCUS use demonstrated significantly higher perception scores. However, because of the cross-sectional study design, it cannot be determined whether frequent POCUS use leads to more positive perceptions or whether physicians with more positive perceptions are more likely to use POCUS frequently.

The high utilization of POCUS observed in the present study reflects its growing integration into contemporary emergency medicine practice. International guidelines increasingly recommend protocol-based POCUS as part of the initial assessment of patients presenting with hypotension or undifferentiated shock because it provides immediate bedside information that can facilitate early diagnosis and guide resuscitation [6]. In our study, the RUSH protocol was the most commonly used protocol for shock assessment. This finding is consistent with the original description of the RUSH examination by Perera et al., who proposed a structured evaluation of the “pump,” “tank,” and “pipes” to rapidly differentiate the major categories of shock [10]. Furthermore, subsequent studies have demonstrated that the RUSH protocol improves diagnostic accuracy in patients with undifferentiated shock, further supporting its widespread adoption in emergency departments [13].

Physicians’ perceptions toward POCUS

Emergency physicians in the present study demonstrated overwhelmingly positive perceptions toward POCUS, with most participants agreeing that it improves diagnostic accuracy, shortens clinical decision-making time, and should be considered an essential competency in emergency medicine. These findings are consistent with previous studies reporting favorable attitudes toward POCUS among emergency physicians and trainees, where bedside ultrasound was widely recognized as an important clinical skill and an integral component of emergency practice [9]. The high level of agreement regarding the incorporation of POCUS into emergency medicine training also reflects the increasing emphasis placed on ultrasound education within residency programs and continuing professional development.

Interestingly, although participants expressed a high level of confidence in using POCUS, fewer participants agreed that it could reduce reliance on advanced imaging modalities. This finding may reflect the complementary role of POCUS in emergency medicine rather than its use as a replacement for comprehensive imaging. In many clinical situations, bedside ultrasound is primarily used to facilitate rapid initial assessment and guide immediate management, while confirmatory imaging remains necessary depending on the patient’s clinical condition and institutional practice [6,10]. Therefore, POCUS should be viewed as an extension of the clinical examination that enhances, rather than replaces, conventional diagnostic investigations.

Perceived benefits of POCUS

Participants identified faster diagnosis as the most important benefit of POCUS, followed by increased physician confidence, improved patient outcomes, and a reduced need for invasive procedures. Specifically, faster diagnosis was reported by 141 (92.8%) participants, increased physician confidence by 88 (57.9%) participants, improved patient outcomes by 62 (40.8%) participants, and a reduced need for invasive procedures by 58 (38.2%) participants. These findings are consistent with previous studies demonstrating that bedside ultrasound provides rapid diagnostic information, allowing emergency physicians to identify the underlying cause of shock promptly and initiate appropriate management without unnecessary delay [6,10]. Early integration of POCUS into the assessment of critically ill patients has been shown to improve diagnostic efficiency and facilitate more timely therapeutic interventions, particularly in time-sensitive conditions such as undifferentiated shock [7,13].

Participants also perceived that POCUS contributes to better patient outcomes and reduces the need for invasive procedures. Although our study evaluated physicians’ perceptions rather than clinical outcomes, these findings are supported by previous evidence. A study by Stanley et al. demonstrated that systematic POCUS in resource-limited settings improved diagnostic accuracy and influenced clinical management through earlier diagnosis and more targeted treatment [14]. Shokoohi et al. further demonstrated that the use of bedside ultrasound reduced diagnostic uncertainty, improved diagnostic accuracy, and guided resuscitation in patients presenting to the emergency department with undifferentiated hypotension [15]. Collectively, these findings reinforce the value of POCUS as an important adjunct to clinical assessment rather than a replacement for comprehensive diagnostic evaluation.

Barriers to POCUS implementation

Despite the favorable perceptions toward POCUS, several barriers to its routine implementation were identified in the present study. Time constraints in the emergency department were the most commonly reported barrier (93 (61.2%)), followed by limited access to ultrasound machines (82 (54.0%)) and lack of formal POCUS training (78 (51.3%)).

Although the survey did not specifically define these barriers, time constraints likely reflect heavy clinical workload and competing demands in busy emergency departments, whereas limited access to ultrasound machines may indicate that equipment was not consistently available when needed for patient assessment.

These findings are consistent with previous studies conducted both internationally and within Saudi Arabia, where insufficient time, inadequate equipment availability, and limited structured training have consistently been identified as major obstacles to the widespread implementation of bedside ultrasound in emergency practice [9,16,17].

Lack of confidence in image interpretation was also reported by 61 (40.1%) participants. Although overall confidence in POCUS was generally high, this finding suggests that competency may vary according to the level of training and frequency of clinical exposure. Previous studies have similarly demonstrated that structured ultrasound education, supervised hands-on practice, and continuous competency assessment are essential for improving physicians’ confidence and encouraging routine POCUS utilization [9,17]. Collectively, these findings indicate that successful implementation of POCUS requires not only adequate equipment but also sustained educational initiatives and institutional support to facilitate its integration into routine emergency care.

Factors associated with POCUS utilization and perception

The present study identified several factors associated with POCUS utilization among emergency physicians. Frequent POCUS use was significantly associated with hospital type and employment in a tertiary care center. Physicians working in university-affiliated training centers reported the highest frequency of POCUS use (22 (95.7%)), followed by those working in Ministry of Health hospitals (48 (69.6%)), whereas physicians working in military and other governmental hospitals reported the lowest frequency (17 (40.5%)). Similarly, 77 (68.1%) participants working in tertiary care centers reported frequent POCUS use compared with 19 (48.7%) participants working in non-tertiary care centers. These findings may reflect differences in institutional resources, access to ultrasound equipment, case mix, and opportunities for ultrasound training across healthcare settings. Tertiary care centers often manage a greater proportion of critically ill patients and may therefore provide more opportunities for routine POCUS use in emergency practice. However, because institutional characteristics were not directly evaluated in the present study, these explanations should be interpreted cautiously. Further research is needed to better understand the institutional factors influencing POCUS utilization across different healthcare settings.

Interestingly, no significant associations were observed between frequent POCUS use and participants’ age, gender, years of emergency medicine experience, or current professional position. This finding suggests that institutional practice environment may have a greater influence on POCUS utilization than individual demographic characteristics alone.

Regarding physicians’ perceptions, higher overall perception scores were significantly associated with years of emergency medicine experience and frequent POCUS use. Participants with 6-10 years of experience demonstrated the highest perception scores, while physicians who reported frequent POCUS use also had significantly more positive perceptions than less frequent users. Conversely, no significant differences in perception scores were observed according to gender, age, professional position, hospital type, or employment in a tertiary care center, suggesting that positive attitudes toward POCUS are broadly shared among emergency physicians regardless of demographic or workplace characteristics.

Strengths and limitations

This study has several strengths. To our knowledge, it is among the few studies in Saudi Arabia specifically evaluating the utilization of POCUS for the diagnosis of shock among emergency physicians across different levels of practice, including consultants, specialists, and senior residents, providing a comprehensive overview of current practice. Furthermore, the questionnaire demonstrated excellent internal consistency, supporting the reliability of the collected data.

In addition, participants were recruited from a variety of healthcare settings, including Ministry of Health, military, university-affiliated, and private hospitals, which enhanced the diversity of the study sample.

Nevertheless, several limitations should be acknowledged. First, the cross-sectional design precludes establishing causal relationships between participant characteristics and POCUS utilization. Second, the use of a non-probability convenience sampling strategy may have introduced selection bias and may limit the generalizability of the findings beyond Riyadh or to emergency physicians practicing in other healthcare settings. Third, the study relied on self-reported responses, which are subject to recall and social desirability bias. Additionally, baseline POCUS training, certification, and objective competency were not assessed. These factors may have influenced participants’ reported utilization patterns, confidence, and perceptions and therefore represent potential sources of residual confounding. Finally, although the study evaluated physicians’ perceptions and reported patterns of POCUS use, it did not assess image acquisition skills, diagnostic accuracy, or patient-centered clinical outcomes. Future studies incorporating objective performance assessments and multicenter prospective designs would provide a more comprehensive evaluation of POCUS implementation in emergency medicine.

Clinical implications and future directions

The findings of the present study have several important implications for emergency medicine practice. The widespread use of POCUS and the positive perceptions reported by participants suggest that POCUS may continue to have an important role in the routine assessment of patients presenting with shock. However, the persistence of barriers such as time constraints, limited access to ultrasound equipment, and insufficient formal training indicates that further efforts are required to optimize its implementation. Expanding structured POCUS training within emergency medicine residency programs, increasing opportunities for supervised hands-on practice, and ensuring adequate access to ultrasound equipment may facilitate broader adoption of POCUS in emergency medicine practice. In addition, institutional policies that support the integration of protocol-based ultrasound into emergency department workflows may further enhance its utilization.

Future studies should move beyond physicians’ perceptions and evaluate whether educational and institutional interventions lead to measurable improvements in POCUS competency, utilization, and patient-centered outcomes.

## Conclusions

This study found that POCUS was widely used by emergency physicians in Riyadh and was perceived as a valuable tool for assessing patients with shock. Most participants reported positive attitudes toward its use, particularly for improving diagnostic accuracy and supporting rapid clinical decision-making. The RUSH protocol was the most commonly utilized approach, highlighting its established role in emergency practice.

Despite these positive findings, barriers including time constraints, limited access to ultrasound machines, and gaps in formal training continue to affect routine POCUS use. Physicians working in tertiary care centers and certain hospital settings reported more frequent POCUS use. Although these findings suggest that institutional support, improved access to ultrasound equipment, and structured training programs may facilitate broader implementation of POCUS, this interpretation should be considered in light of the cross-sectional study design, which does not permit causal inference.

## Appendices

Appendix 1: Survey questionnaire

Point-of-Care Ultrasound (POCUS) in Shock Diagnosis: Perceptions, Usage, and Barriers Among Emergency Physicians in Riyadh

Section 1: Consent

Background: Shock is a critical and potentially life-threatening condition encountered in emergency departments. Point-of-care ultrasound (POCUS) is an important bedside tool to guide early evaluation and management. However, its use varies due to differences in training, equipment, and institutional support.

Purpose: You are invited to participate in this survey about the use of point-of-care ultrasound (POCUS) in the evaluation of shock in emergency departments. Participation is voluntary, and responses are anonymous. No personal identifiers will be collected.

1. Do you agree to participate in this survey?

☐ Yes ☐ No

Section 2: Demographics

2. Age group

☐ <30 ☐ 31-39 ☐ 40-49 ☐ >50

3. Gender

☐ Male ☐ Female

4. Years of experience in emergency medicine

☐ 0-5 years ☐ 6-10 years ☐ >10 years

5. Current position

☐ Senior Emergency Medicine Resident ☐ Emergency Medicine Specialist ☐ Emergency Medicine Consultant

6. Type of hospital

☐ Ministry of Health (MOH) ☐ Military ☐ Private ☐ University-affiliated training center ☐ Other: 

7. Do you work in a tertiary care center?

☐ Yes ☐ No

Section 3: POCUS Utilization

8. How often do you use POCUS in the diagnosis of shock?

☐ Never ☐ Rarely (1-2 times/month) ☐ Occasionally (1-2 times/week) ☐ Frequently (3-5 times/week) ☐ Daily

9. Which POCUS protocols do you typically use for shock evaluation? (Check all that apply.)

☐ RUSH ☐ SHoC ☐ FAST ☐ Other: 

10. For which clinical conditions do you most commonly use POCUS in the context of shock? (Check all that apply.)

☐ Undifferentiated shock ☐ Trauma ☐ Sepsis ☐ Cardiac arrest ☐ Pulmonary embolism ☐ Hypovolemic shock ☐ Cardiogenic shock ☐ Other: 

Section 4: Perceptions

11. Please indicate your level of agreement with the following statements (1 = Strongly Disagree, 2 = Disagree, 3 = Neutral, 4 = Agree, 5 = Strongly Agree).

• POCUS improves diagnostic accuracy in shock management. • POCUS reduces the time to clinical decision-making. • POCUS should be a mandatory skill in emergency medicine training. • POCUS use decreases reliance on advanced imaging (e.g., CT/MRI). • I feel confident in my ability to use POCUS effectively. Response scale: 1  2  3  4  5

12. What do you consider the most important benefits of using POCUS in shock diagnosis? (Check all that apply.)

☐ Faster diagnosis ☐ Improved patient outcomes (e.g., reduced mortality, shorter ED stays) ☐ Reduced need for invasive procedures ☐ Increased physician confidence ☐ Other:

Section 5: Barriers to POCUS Use

13. What are the main barriers preventing routine POCUS use in your practice? (Check all that apply.)

☐ Lack of formal training ☐ Lack of confidence in interpretation ☐ Limited access to ultrasound machines ☐ Time pressure during emergency care ☐ Lack of institutional support (e.g., funding, administrative policies, mentorship) ☐ Other: 

14. What do you believe would most improve POCUS adoption in emergency medicine? (Check all that apply.)

☐ Mandatory residency training ☐ Hands-on workshops ☐ Increased availability of ultrasound machines ☐ Institutional policies supporting POCUS use ☐ Other: 

15. Do you have any additional comments or suggestions regarding POCUS training or usage in emergency medicine?
